# Draft Genome Sequence of *Frankia* sp. Strain BCU110501, a Nitrogen-Fixing Actinobacterium Isolated from Nodules of *Discaria trinevis*

**DOI:** 10.1128/genomeA.00503-13

**Published:** 2013-07-11

**Authors:** Luis G. Wall, Nicholas Beauchemin, Michael N. Cantor, Eugenia Chaia, Amy Chen, J. Chris Detter, Teal Furnholm, Faten Ghodhbane-Gtari, Lynne Goodwin, Maher Gtari, Cliff Han, James Han, Marcel Huntemann, Susan Xinyu Hua, Natalia Ivanova, Nikos Kyrpides, Victor Markowitz, Kostas Mavrommatis, Natalia Mikhailova, Henrik P. Nordberg, Imen Nouioui, Galina Ovchinnikova, Ioanna Pagani, Amrita Pati, Arnab Sen, Saubashya Sur, Ernest Szeto, Subarna Thakur, Chia-Lin Wei, Tanja Woyke, Louis S. Tisa

**Affiliations:** University of Quilmes, Bernal, Argentinaa; University of New Hampshire, Durham, New Hampshire, USAb; Los Alamos National Laboratory, Los Alamos, New Mexico, USAc; Centro Regional Universitario, Bariloche, Argentinad; Department of Energy (DOE) Joint Genome Institute, Walnut Creek, California, USAe; University of Tunis–El Manar, Tunis, Tunisiaf; University of North Bengal, Siliguri, Indiag

## Abstract

*Frankia* forms a nitrogen-fixing symbiosis with actinorhizal plants. We report a draft genome sequence for *Frankia* sp. strain BCU110501, a nitrogen-fixing actinobacterium isolated from nodules of *Discaria trinevis* grown in the Patagonia region of Argentina.

## GENOME ANNOUNCEMENT

The genus *Frankia* forms a symbiotic nitrogen-fixing association with woody trees and shrubs, resulting in a root nodule formation (1–3). *Frankia* exists either in a free-living state in the soil or in symbiosis with actinorhizal plants. These actinorhizal plants comprise over 200 different plant species belonging to 8 distinct plant families. Because of symbiosis, these globally distributed actinorhizal plants are able to colonize harsh environmental terrains under diverse ecological conditions. Phylogenetic studies have shown that *Frankia* has four major lineages or clusters that can also be defined by physiology, host specificity, and mode of infection of the host plant (4–7). Genomes for representatives from each of these clusters have been sequenced (8–13) and have provided essential baseline information for genomic approaches toward understanding these novel bacteria.

*Frankia* strains from cluster III have the greatest metabolic versatility, are known to associate with five plant families (*Betulaceae*, *Myricaceae*, *Elaeagnaceae*, *Rhamnaceae*, and *Casuarinaceae*), and are considered broad-host-range symbionts. Many of these strains have adapted to harsh environmental conditions. *Frankia* sp. strain BCU110501 (also called Dtl1 and T1) was isolated from a field nodule of *Discaria trinevis* growing wild in a semiarid steppe (40°41′S, 71°10′W) of the Argentinian Reserve and National Park “Parque Nacional Nahuel Huapi,” located in the northwest of Patagonia (14). The physiology and infectivity of this strain have been well studied (15–20), and it uses an intercellular route of plant infection (18). *Frankia* sp. strain BCU110501 was chosen for sequencing as another cluster III representative with broad-host-range properties and an intercellular infection route. Strain BCU110501 was sequenced to provide information about the potential ecological roles of the *Frankia* strains and their interactions with actinorhizal plants.

The draft genome of *Frankia* sp. strain BCU110501 was generated at the Department of Energy (DOE) Joint Genome Institute (JGI) using Illumina technology (21). An Illumina standard shotgun library was constructed and sequenced using the Illumina HiSeq 2000 platform, which generated 12,553,550 reads totaling 1,883.0 Mbp. All techniques for DNA isolation, library construction, and sequencing were performed according to JGI standards and protocols (http://www.jgi.doe.gov). The Illumina sequence data were assembled using Velvet (version 1.1.04) (22) and Allpaths-LG (version r41043) (23). The final draft assembly contained 207 contigs in 207 scaffolds. The total size of the genome is 7.9 Mbp and the final assembly is based on 950.8 Mbp of Illumina data, which provides an average 120.4× coverage of the genome.

The draft genome of *Frankia* BCU110501 was resolved to 207 scaffolds consisting of 7,891,711 bp with a G+C content of 72.39%, 6,742 candidate protein-encoding genes, 47 tRNA genes, and 2 rRNA regions.

### Nucleotide sequence accession numbers.

The *Frankia* sp. strain BCU110501 genome sequence has been deposited at DDBJ/EMBL/GenBank under the accession number ARDT00000000. The version described in this paper is the first version, ARDT01000000.

## References

[1] Benson DR Silvester WB 1993 Biology of *Frankia* strains, actinomycete symbionts of actinorhizal plants. Microbiol. Rev. 57:293–319 8336669 10.1128/mr.57.2.293-319.1993 PMC372911

[2] Schwencke J Caru M 2001 Advances in actinorhizal symbiosis: host plant-*Frankia* interactions, biology, and applications in arid land reclamation. A review. Arid Land Res. Manag. 15:285–327

[3] Chaia EE Wall LG Huss-Danell K 2010 Life in soil by actinorhizal root nodule endophyte *Frankia*. A review. Symbiosis 51:201–226

[4] Benson DR Clawson ML 2000 Evolution of the actinorhizal plant symbioses, p 207–224 *In* Triplett EW, Prokaryotic nitrogen fixation: a model system for analysis of biological process. Horizon Scientific Press, Wymondham, United Kingdom

[5] Normand P Orso S Cournoyer B Jeannin P Chapelon C Dawson JO Evtushenko L Mirsra AK 1996 Molecular phylogeny of the genus *Frankia* and related genera and emendation of the family *Frankiaceae*. Int. J. Syst. Bacteriol. 46:1–9 8573482 10.1099/00207713-46-1-1

[6] Nouioui I Ghodhbane-Gtari F Beauchemin NJ Tisa LS Gtari M 2011 Phylogeny of members of the *Frankia* genus based on *gyrB*, *nifH* and *glnII* sequences. Antonie Van Leeuwenhoek 100:579–587 21713368 10.1007/s10482-011-9613-y

[7] Ghodhbane-Gtari F Nouioui I Chair M Boudabous A Gtari M 2010 16S-23S rRNA intergenic spacer region variability in the genus *Frankia*. Microb. Ecol. 60:487–495 20179918 10.1007/s00248-010-9641-6

[8] Normand P Lapierre P Tisa LS Gogarten JP Alloisio N Bagnarol E Bassi CA Berry AM Bickhart DM Choisne N Couloux A Cournoyer B Cruveiller S Daubin V Demange N Francino MP Goltsman E Huang Y Kopp OR Labarre L Lapidus A Lavire C Marechal J Martinez M Mastronunzio JE Mullin BC Niemann J Pujic P Rawnsley T Rouy Z Schenowitz C Sellstedt A Tavares F Tomkins JP Vallenet D Valverde C Wall LG Wang Y Medigue C Benson DR 2007 Genome characteristics of facultatively symbiotic *Frankia* sp. strains reflect host range and host plant biogeography. Genome Res. 17:7–15 17151343 10.1101/gr.5798407 PMC1716269

[9] Normand P Queiroux C Tisa LS Benson DR Rouy Z Cruveiller S Medigue C 2007 Exploring the genomes of *Frankia*. Physiol. Plant. 130:331–343

[10] Persson T Benson DR Normand P Vanden Heuvel B Pujic P Chertkov O Teshima H Bruce DC Detter C Tapia R Han S Han J Woyke Pitlock S Pennacchio L Nolan M Ivanova N Pati A Land ML Pawlowski K Berry AM 2011 Genome sequence of “*Candidatus* Frankia datiscae” Dg1, the uncultured microsymbiont from nitrogen-fixing root nodules of the dicot *Datisca glomerata*. J. Bacteriol. 193:7017–7018 22123767 10.1128/JB.06208-11 PMC3232863

[11] Ghodbhane-Gtari F Beauchemin N Bruce D Chain P Chen A Walston Davenport K Deshpande S Detter C Furnholm T Goodwin L Gtari M Han C Han J Huntemann M Ivanova N Kyrpides N Land ML Markowitz V Mavrommatis K Nolan M Nouioui I Pagani I Pati A Pitluck S Santos CL Sen A Sur S Szeto E Tavares F Teshima H Thakur S Wall LG Woyke T Tisa LS 2013 Draft genome sequence of *Frankia* sp. strain CN3, an atypical, noninfective (Nod^–^) ineffective (Fix^–^) isolate from *Coriaria nepalensis*. Genome Announc. 1(2):e00085-13. 10.1128/genomeA.00085-13 23516212 PMC3622958

[12] Sen A Beauchemin N Bruce D Chain P Chen A Walston Davenport K Deshpande S Detter C Furnholm T Ghodbhane-Gtari F Goodwin L Gtari M Han C Han J Huntemann M Ivanova N Kyrpides N Land ML Markowitz V Mavrommatis K Nolan M Nouioui I Pagani I Pati A Pitluck S Santos CL Sur S Szeto E Tavares F Teshima H Thakur S Wall L Woyke T Wishart J Tisa LS 2013 Draft genome sequence of *Frankia* sp. strain QA3, a nitrogen-fixing actinobacterium isolated from the root nodule of *Alnus nitida*. Genome Announc. 1(2):e00103-13. 10.1128/genomeA.00103-13 23516220 PMC3622976

[13] Nouioui I Beauchemin N Cantor MN Chen A Detter JC Furnholm T Ghodhbane-Gtari F Goodwin L Gtari M Han C Han J Huntemann M Hua SX Ivanova N Kyrpides N Markowitz V Mavrommatis K Mikhailova N Nordberg HP Ovchinnikova G Pagani I Pati A Sen A Sur S Szeto E Thakur S Wall L Wei C-L Woyke T Tisa LS 2013 Draft genome sequence of *Frankia* sp. strain BMG5.12, a nitrogen-fixing actinobacterium isolated from Tunisian soils. Genome Announc. 1(4):e00468-13. 10.1128/genomeA.00468-13 23846272 PMC3709149

[14] Chaia E 1998 Isolation of an effective strain of *Frankia* from nodules of *Discaria trinervis* (*Rhamnaceae*). Plant Soil 205:99–102

[15] Chaia EE Solans M Vobis G Wall LG 2007 Infectivity variation of *Discaria trinervis*-nodulating *Frankia* in Patagonian soil according to season and storage conditions. Physiol. Plant. 130:357–360

[16] Gabbarni LA Wall LG 2008 Analysis of nodulation kinetics in *Frankia-Discaria trinervis* symbiosis reveals different factors involved in the nodulation process. Physiol. Plant. 133:776–778 18397207 10.1111/j.1399-3054.2008.01096.x

[17] Valverde C Ferrari A Wall LG 2002 Phosphorus and the regulation of nodulation in the actinorhizal symbiosis, between *Discaria trinervis* (*Rhamnaceae*) and *Frankia* BCU11050. New Phytol. 153:43–52

[18] Valverde C Wall LG 1999 Time course of nodule development in *Discaria trinervis* (*Rhamnaceae*)-*Frankia* symbiosis. New Phytol. 141:345–354 10.1046/j.1469-8137.1999.00345.x 33862924

[19] Valverde C Wall LG 1999 Regulation of nodulation in *Discaria trinervis* (*Rhamnaceae*)-*Frankia* symbiosis. Can. J. Bot. 77:1302–1310 10.1046/j.1469-8137.1999.00345.x 33862924

[20] Valverde C Ferrari A Wall LG 2009 Effect of calcium in the nitrogen-fixing symbiosis between actinorhizal *Discaria trinervis* (*Rhamnaceae*) and *Frankia*. Symbiosis 49:151–155

[21] Bennett S 2004 Solexa Ltd. Pharmacogenomics 5:433–438 15165179 10.1517/14622416.5.4.433

[22] Zerbino D Birney E 2008 Velvet: algorithms for de novo short read assembly using de Bruijn graphs. Genome Res. 18:821–829 18349386 10.1101/gr.074492.107 PMC2336801

[23] Gnerre S MacCallum I 2011 High–quality draft assemblies of mammalian genomes from massively parallel sequence data. Proc. Natl. Acad. Sci. U. S. A. 108:1513–1518 21187386 10.1073/pnas.1017351108 PMC3029755

